# Unraveling the structure of sugarcane bagasse after soaking in concentrated aqueous ammonia (SCAA) and ethanol production by *Scheffersomyces* (*Pichia*) *stipitis*

**DOI:** 10.1186/1754-6834-6-102

**Published:** 2013-07-15

**Authors:** Anuj K Chandel, Felipe AF Antunes, Messias B Silva, Silvio Silvério da Silva

**Affiliations:** 1 Department of Biotechnology, Engineering School of Lorena, University of São Paulo, Lorena 116-12.602.810, Sao Paulo, Brazil; 2 Department of Chemical Engineering, Engineering School of Lorena, University of São Paulo, Lorena 12.602.810, Sao Paulo, Brazil

**Keywords:** Sugarcane bagasse, Taguchi design, Soaking in concentrated aqueous ammonia, *Scheffersomyces (Pichia) stipitis*, Bioethanol

## Abstract

**Background:**

Fuel ethanol production from sustainable and largely abundant agro-residues such as sugarcane bagasse (SB) provides long term, geopolitical and strategic benefits. Pretreatment of SB is an inevitable process for improved saccharification of cell wall carbohydrates. Recently, ammonium hydroxide-based pretreatment technologies have gained significance as an effective and economical pretreatment strategy. We hypothesized that soaking in concentrated aqueous ammonia-mediated thermochemical pretreatment (SCAA) would overcome the native recalcitrance of SB by enhancing cellulase accessibility of the embedded holocellulosic microfibrils.

**Results:**

In this study, we designed an experiment considering response surface methodology (Taguchi method, L_8_ orthogonal array) to optimize sugar recovery from ammonia pretreated sugarcane bagasse (SB) by using the method of soaking in concentrated aqueous ammonia (SCAA-SB). Three independent variables: ammonia concentration, temperature and time, were selected at two levels with center point. The ammonia pretreated bagasse (SCAA-SB) was enzymatically hydrolysed by commercial enzymes (Celluclast 1.5 L and Novozym 188) using 15 FPU/g dry biomass and 17.5 Units of β-glucosidase/g dry biomass at 50°C, 150 rpm for 96 h. A maximum of 28.43 g/l reducing sugars corresponding to 0.57 g sugars/g pretreated bagasse was obtained from the SCAA-SB derived using a 20% v/v ammonia solution, at 70°C for 24 h after enzymatic hydrolysis. Among the tested parameters, pretreatment time showed the maximum influence (p value, 0.053282) while ammonia concentration showed the least influence (p value, 0.612552) on sugar recovery. The changes in the ultra-structure and crystallinity of native SCAA-SB and enzymatically hydrolysed SB were observed by scanning electron microscopy (SEM), x-ray diffraction (XRD) and solid-state ^13^C nuclear magnetic resonance (NMR) spectroscopy. The enzymatic hydrolysates and solid SCAA-SB were subjected to ethanol fermentation under separate hydrolysis and fermentation (SHF) and simultaneous saccharification and fermentation (SSF) by *Scheffersomyces* (*Pichia*) *stipitis* NRRL Y-7124 respectively. Higher ethanol production (10.31 g/l and yield, 0.387 g/g) was obtained through SSF than SHF (3.83 g/l and yield, 0.289 g/g).

**Conclusions:**

SCAA treatment showed marked lignin removal from SB thus improving the accessibility of cellulases towards holocellulose substrate as evidenced by efficient sugar release. The ultrastructure of SB after SCAA and enzymatic hydrolysis of holocellulose provided insights of the degradation process at the molecular level.

## Background

The increased demand for energy, regular price hikes of gasoline, geo-political factors, and environmental damage have alarmed the scientific community, economists and governments to evaluate the potential of cellulosic ethanol as a sustainable, economic and ecofriendly alternative of gasoline [1-3]. Sugarcane bagasse (SB) is an excellent raw material for second generation ethanol production in countries like Brazil, India and China where it is generated in huge amount every year [2]. In Brazil, around 163–169 million tons of SB was generated in the 2012/13 harvest [4]. Almost 50% of SB is used for energy generation in industry but the rest remains unused. The judicious conversion of the left over SB into second generation ethanol may have sustainable, economic and strategic benefits; however it needs intensive technological and multidisciplinary efforts [1]. SB is primarily comprised of carbohydrate polymers (hemicellulose and cellulose) and lignin linked into a highly complex and recalcitrant matrix. Because of this, pretreatment is an inevitable process to render the carbohydrate fraction accessible for cellulolytic enzyme action to release fermentable sugars followed by their bioconversion into ethanol by microbial fermentation. Together, pretreatment and saccharification contributes 50-60% of the total cost incurred for bioethanol production from lignocellulosics [3]. In the past, several pretreatment methods such as auto-hydrolysis, liquid hot water, super critical fluids, alkali, acid, organic solvents, and biological pretreatments have been applied to SB [4] to make it more amenable to saccharification. Despite several developments in pretreatment processes for SB, complete mechanisms involved in biomass deconstruction remain to be elucidated thus complicating the decision of which pretreatment to apply [4].

Ammonium hydroxide mediated pretreatment is a state-of-the-art technology which specifically acts on the lignin-carbohydrate complex by cleaving ester linkages via ammonolysis to remove lignin and increase the accessibility of the remaining holocellulose fraction to cellulase action [5]. Ammonia pretreatment in any form such as ammonia fiber expansion (AFEX), ammonia freeze explosion and soaking in aqueous ammonia (SAA) has been proven as an effective strategy for the delignification of various lignocellulosic substrates [5-13]. Key for the success of AFEX is selection of influential parameters in their appropriate ranges to maximize lignin removal while minimizing carbohydrate degradation [5,9]. After pretreatment, the ammonia solution can be recovered for subsequent use as a delignification agent to further economize the process [5].

The Taguchi method is a statistical method that utilizes the fundamental principles of statistics, randomization and replication for the design and analysis of factorial experiments. This method allows the investigation of the influence of several parameters on a process while saving time by reducing the number of experiments required [14].

The delignified material can be separately hydrolysed by cellulolytic enzymes and the resultant sugar syrup can be fermented directly into ethanol by an appropriate microorganism (SHF). Another option is to perform the enzymatic hydrolysis and fermentation of all released sugars (pentose and hexose) into ethanol simultaneously (SSF). The latter has shown several advantages such as process intensification, less processing time, low contamination risk, cost reduction and high ethanol titers [15]. The most important feature of SSF is to overcome the enzyme inhibition by the released sugars during enzymatic hydrolysis as the released sugars are simultaneously converted into ethanol by the microorganism. However, the difference in optimum temperature for microbial fermentation and enzymatic hydrolysis is a major concern of SSF [3,15].

Here, we report the ammonia pretreatment optimization of SB and the efficiency of all pretreated bagasse samples for sugar recovery during enzymatic hydrolysis. Ethanol production was evaluated under SHF and SSF using the sugar hydrolysate of optimized ammonia pretreated bagasse or delignified SB by *S. stipitis* NRRL Y-7124. Structural and crystallinity changes in native, ammonia pretreated and enzymatically digested SB were also analyzed by SEM, XRD and solid-state ^13^C NMR spectroscopy.

## Results and Discussion

### Optimization of ammonia pretreatment and enzymatic digestibility

Conditions for the ammonia pretreatment process were optimized for the maximum delignification of SB considering three important process variables: ammonia concentration, temperature and time at three different levels. Sugar recovery after enzymatic hydrolysis was the responsive variable (Table 1 and 2). In the Taguchi design of experiments, all process variables or factors are arranged in an orthogonal array. The orthogonal layout facilitates the effect of each variable individually as well as in relation to each other to provide a relative value. The Taguchi L_8_ orthogonal array minimizes the number of test runs required, while keeping the pair-wise balancing property [16]. The influence of each process variable on delignification and hydrolysis efficiency of SB can be characterized using an L_8_ orthogonal experimental array. In the present study, the variables of ammonia concentration, temperature and time and their ranges were chosen as they have previously been shown to affect the delignification process [6,11]. The rate of enzymatic hydrolysis for saccharification primarily depends upon the amount of lignin present in the substrate [17]. However, other parameters such as substrate concentration, enzyme loading, temperature and time also play a role in sugar recovery during enzymatic saccharification of pretreated biomass [3].

**Table 1:** Factors and their levels for soaking in concentrated aqueous ammonia solution (SCAA) of sugarcane bagasse

**Factors**	**Lower level**	**Mid-point**	**Higher level**
Ammonia concentration (% v/v)	20	24	28
Temperature (°C)	50	60	70
Time (h)	8	16	24

**Table 2:** **Taguchi orthogonal array design of experiment with central point (L**_**8**_**) and the data collection**

**Experimental run**	**Ammonia concentration (% v/v)**	**Time (h)**	**Temperature (°C)**	**Recovery of reducing sugars (g/L)**
1	20	8	50	14.27
2	28	8	50	18.51
3	20	24	50	21.97
4	28	24	50	22.87
5	20	8	70	22.31
6	28	8	70	21.12
7	20	24	70	28.43
8	28	24	70	25.48
9	24	16	60	22.87

**Figure 1:**
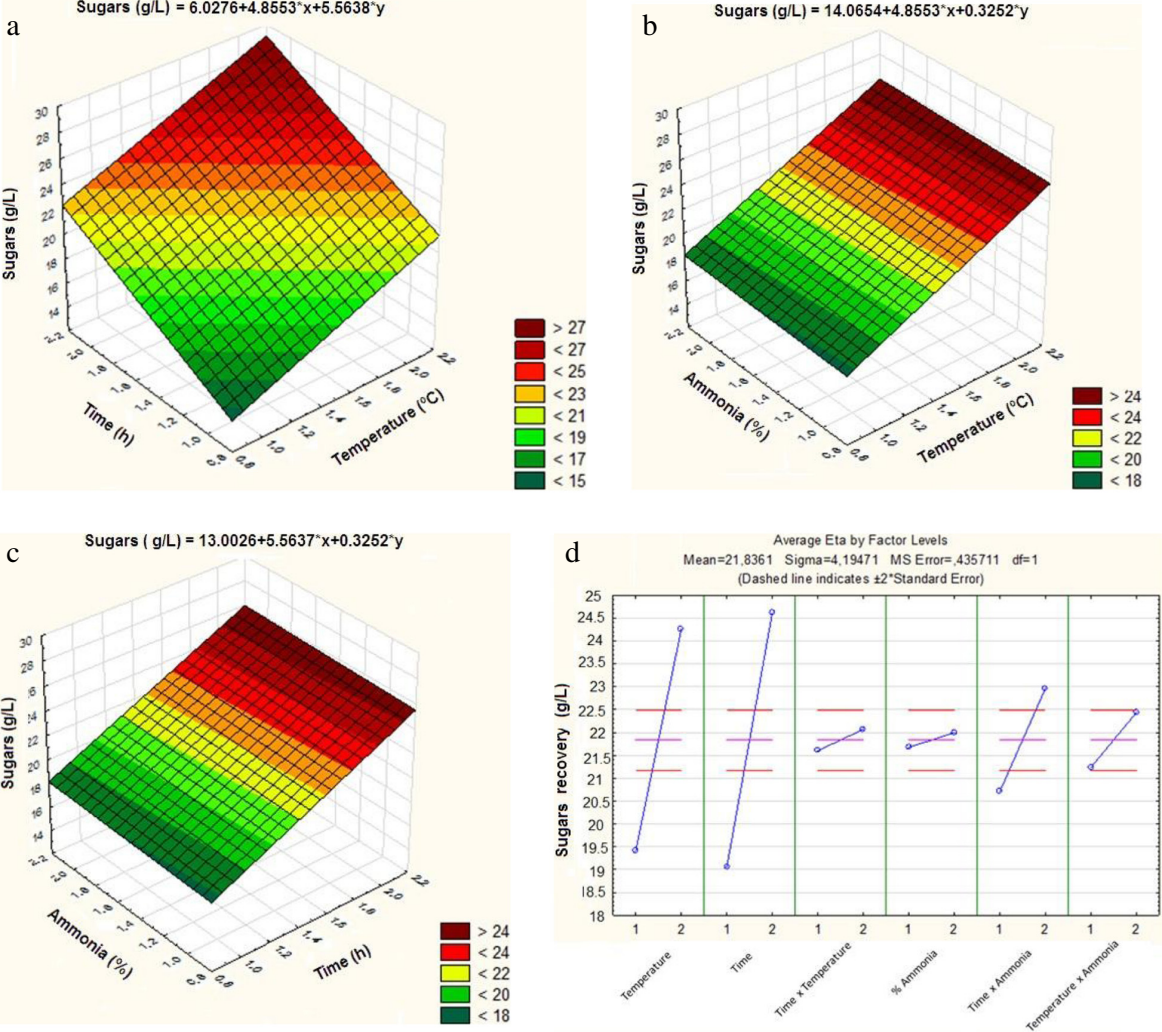
Statistical analysis of SCAA-SB showing the perspective plots fitted between process variables (a) Pretreatment time (h) and temperature (°C) (b) Ammonium hydroxide concentration (% v/v) and temperature (°C) (c) Ammonium hydroxide concentration (% v/v) and pretreatment time (h) (d) Effect of individual variables and their interaction on total reducing sugars recovery (g/l).

The Taguchi L_8_ orthogonal array revealed significant variation in sugar recovery from the ammonia-pretreated substrate after enzymatic hydrolysis (Table 2). Maximum sugar recovery was determined to be 28.43 g/l, 0.57 g/g bagasse after 72 h of enzymatic hydrolysis in experimental run 7 where 20% (v/v) ammonia at 70°C with a 24 h pretreatment time were used for delignification. The lowest sugar recovery, 14.27 g/l, was obtained from experimental run 1 (20% v/v NH_4_OH, 8 h, 50°C) after 72 h of enzymatic hydrolysis. Statistical analysis of the enzymatic hydrolysis of SCAA-SB substrate shows the perspective plots of fitted between process variables (Figure 1a, b and c). The data shows that time and temperature of ammonia pretreatment plays a crucial role in delignification (Figure 1d). Figure 1 (a, b and c) shows the response surface analysis for total sugar recovery from SCAA-SB after enzymatic hydrolysis. Figure 1 (d) shows the effect of each variable and their possible interaction towards the responsive variable, total reducing sugars released after enzyme hydrolysis. Table 3 presents the effect of each variable and their interaction on sugar recovery. Temperature and pretreatment time at individual levels showed a maximum sum of squares (SS) compared to other variables. Pretreatment time showed major influence (p value, 0.05382) followed by temperature (p value, 0.061013). Alone, ammonia concentration did not have a major influence on sugars recovery. Among the interactive effects of two different variables, time and ammonia concentration was more effective than other parameters at interactive levels (Figure 1d).

**Table 3:** Analysis of variance (ANOVA), F-test and interactions

**Variables**	**SS**	**df**	**MS**	**F**	**p**
Temperature (°C)	47.14690	1	47.14690	108.2068	0.061013
Time (h)	61.91063	1	61.91063	142.0910	0.053282
Time × Temperature	0.43665	1	0.43665	1.0021	0.499659
Ammonia concentration (% v/v)	0.21158	1	0.21158	0.4856	0.612552
Time × Ammonia concentration	10.13175	1	10.13175	23.2534	0.130174
Temperature x Ammonia concentration	2.89562	1	2.89562	6.6457	0.235574
Residual	0.43571	1	0.43571		

*SS* sum of square, *df* degree of freedom, *MS* mean square, *F* Statistical test (explained variance/unexplained variance), *p* probability. Effect of all three process variables (Temperature, pretreatment time and ammonium hydroxide concentration) and their interaction on sugars recovery. Temperature and pretreatment time at individual level showed maximum sum of squares (SS) compared to other variables. Pretreatment time showed major influence (p value, 0.05382) followed by temperature (p value, 0.061013). Ammonia concentration at individual level did not present major influence on sugars recovery.

In addition to appropriate hydrolysis conditions, a fine cocktail of cellulolytic enzymes comprising cellulases, β-glucosidases, xylanases is required to obtain sugar monomers from SCAA-SB [3]. We obtained low sugar recovery from ammonia-pretreated bagasse samples probably due to low solid (SB) loadings in ammonia solutions (1:10). Ammonium hydroxide solubilizes the hemicellulose and cellulose considerably in addition to significant removal of lignin from the substrate [10]. For the pretreatment of SB, process parameters such as ammonia concentration, temperature and time that were used in this study were previously studied with various lignocellulosic materials [7,13,18]. The important aspect of AFEX pretreatment at mild temperatures (40-90°C) for longer reaction times is known to preserve most of the carbohydrate in the samples [12]. For instance, rice straw after pretreatment with aqueous ammonia (21% w/w, 10 h, 69°C) showed 71.1% enzymatic hydrolysis using 15 FPU of cellulase/g-glucan and 30 CBU of β-glucosidase/g-glucan). The sugar recovery from various AFEX-pretreated substrates depends upon the raw material and pretreatment conditions employed. For example, Kim et al. [11] pretreated barley hull (15 wt.% ammonia, 24-72 h, 75°C) and reported 50-66% lignin removal which showed saccharification yield (83% glucan, 63% xylan solubilisation) with 15 FPU/g –glucan enzyme loadings. These ammonia-assisted delignification studies were based on varying range of diluted ammonia solution (15-29% ammonia), temperature (50-90°C), time (12-72 h) and solid to liquid ratio (1: 10).

### Cell wall characterization and structural analysis

#### Cell wall composition

The physico-chemical analysis of native sugarcane bagasse cell wall revealed the following composition (on percentage base): total lignin, 27.53% (24.46% Klason lignin + 3.07% acid soluble lignin); 41.96% cellulose; 19.20% hemicellulose; 2.90% extractives; 8.16% moisture and 1.5% structural ash. This SB composition is comparable with other reports [2,4,18]. However, direct comparison of compositional data is not feasible since the chemical composition of sugarcane bagasse like any other lignocellulosic material depends on several factors such as the variety, location, and agricultural practices used to grow the crop and the employed analytical method for cell wall composition analysis [2]. After pretreatment of SB with 20% (v/v) NH_4_OH for 24 h at 70°C, 41.51% lignin removal was observed. The final cell wall composition (% base) of SCAA-bagasse was as follows: lignin, 16.10; cellulose, 67.42; hemicellulose, 6.0; ash, 2.5 and moisture, 8.16. Extractives were found in negligable amount. Cellulose was found to be increased by 62.20% in SCAA-bagasse samples. On the contrary, a considerable amount of hemicellulose (68.75%) was lost during the pretreatment. Loss of hemicellulose was expected in addition to removal of lignin during the SCAA of sugarcane bagasse. However, loss of hemicellulose and removal of lignin during ammonia hydroxide mediated pretreatment depend upon the reaction conditions and lignocellulosic material used. Prior and Day [18] reported the composition of sugarcane bagasse (21.1% lignin, 56.6% glucan, 24% xylan and 1.2% arabinan) after ammonia hydroxide pretreatment (0.5 g NH_4_OH of 28% v/v per gram bagasse, 160°C, 60 min). Aita et al. [6] observed 55% delignification, 9% glucan loss and 30% removal of hemicellulose from ammonia pretreated (bagassae 1: NH_4_OH 0.5: water 8, 160°C, 60 min, 0.9-1.1 MPa) high fiber sugarcane bagasse. Aqueous ammonium hydroxide pretreatment of barley hull (15% w/w hydrated ammonia, 24-72 h, 75°C) showed 50-66% lignin removal while maintained 65-76% xylan and negligible loss of glucan [11]. Ko et al. [12] reported 60.6 ± 0.3% lignin removal and retaining of 86.9 ± 1.1% glucan in aqueous-ammonia soaking pretreatment of rice straw (70°C, 12 h and 20% w/w hydrated ammonia) which eventually showed 70.4 ± 1.1% enzymatic digestibility. Complete delignification from biomass is extremely difficult due to the mechanistic location of lignin within the lignin-carbohydrate complex, strong poly-ring bonds of C-O-C, C-C and hydrophobicity [6,11]. It is speculated that the aqueous ammonia-mediated pretreatment at low temperature show a considerable loss of hemicellulose and lower lignin removal [11,12] which is also evident with the results obtained in the present study.

### Scanning electron microscopy (SEM)

**Figure 2:**
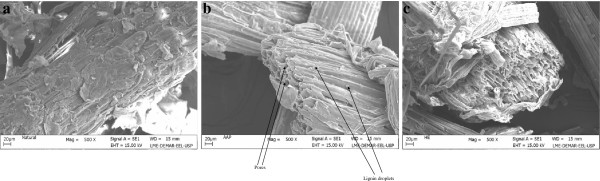
**Scanning electron microscopic (SEM) analysis of SB showing surface images (a) Natural (b) SCAA-SB (c) After enzymatic hydrolysis (EH-SB).** Natural SB showed rough and compact surface with extractives. SCAA-SB showed smooth and disrupted surface with several pores. EH-bagasse showed maximum heterogeneous nature of surface revealing the destruction of cellulose.

The ultra-structure of the SB cell walls was studied using SEM analysis to study the impact of ammonium hydroxide followed by enzymatic digestion. Figure 2 (a) shows the anatomy of native, milled SB which is compact, rough and has thick walled fiber cells interlinked with pith. Fibers are constituted by parallel stripes and are superficially covered with extractives. The most apparent effect of SCAA pretreatment is the change in color of SB (from whitish to light brown). SEM analysis of SCAA-SB proved the physical disruption of fiber and pith with the separation of fibers. Appearance of pores and lignin droplets due to partial delocalization can be seen in ammonia pretreated SB (Figure 2 b). SCAA primarily removes lignin while simultaneously acting as a swelling agent; which aids the accessibility of celluloliscs towards enzymatic action. Removal of lignin in conjunction with other physical changes in the surface morphology of bagasse such as loosening of the matrix, partial degradation of hemicellulose, dismantling of vascular bundles and detaching of fibers, led to the appreciable enzymatic degradation of carbohydrates into their monomer sugar constituents [10,19]. Similar changes were observed in surface images of rice straw pretreated with aqueous ammonia [12] and for sugarcane bagasse pretreated with microwave-alkali [20]. Micrographs of enzymatically hydrolyzed SCAA-bagasse clearly show the destruction of cellulose and the heterogeneous nature of the samples (Figure 2c). The structural changes in the cell wall of sugarcane bagasse after SCAA pretreatment and enzymatic hydrolysis was in accordance with our previous studies with *Saccharum spontaneum*[10].

### X-ray diffraction (XRD)

**Figure 3:**
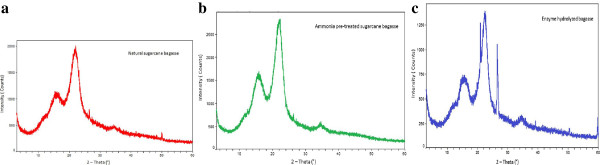
**Spectra show the relationship between the diffraction angle (2-theta) and x-ray intensity (counts).** X-ray diffraction (XRD) pattern of native, ammonia pretreated bagasse (SCAA-SB) and enzyme hydrolysed bagasse (EH-SB). Native SB showed 50.5% crystallinity index. SCAA-SB showed 65.75% crystallinity index because of high cellulose amount in substrate. EH-SB did not show major improvement in crystallinity index (67.25%).

The XRD profile of native, SCAA-bagasse and enzyme digested bagasse is shown in Figure 3 (a, b and c). The crystallinity index (CrI) of all three samples was calculated following the method of Segal et al [21] and Park et al. [22]. The intensities (I_002_) of the amorphous cellulose peak and intensity of crystalline cellulose peak were considered for the CrI calculation. The CrI of native SB, 50.5%, was less than for the ammonia pretreated SB, 65.75%. Enzyme digested SCAA-bagasse had a CrI of 67.25%. The primary reason for the increased crystallinity is the removal of lignin which increases the cellulose concentration in the bagasse compared to native SB. Biomass crystallinity is considered to be an important factor for biomass digestibility [17,22]. Rezende et al. [17] observed a linear increment in CrI of SB after sequential pretreatment with dilute sulfuric acid followed by dilute sodium hydroxide mediated pretreatment due to amelioration in cellulose amount after each pretreatment. Sindhu et al. [23] reported the increased CrI (67.83%) of dilute sulfuric acid + formic acid pretreated sugarcane bagasse samples. They observed higher CrI values than for ammonia-pretreated bagasse probably due to hemicellulose removal in addition to delocalization of lignin. Velmurugan and Muthukumar [24] reported a CrI of 66% for sodium hydroxide pretreated bagasse, which increased to 70.7% after sono-assisted pretreatment as compared to native SB (50%). We did not observe significant changes in CrI (67.24%) of enzyme digested samples of SCAA-SB. The reason for this is not clear. However, Binod et al. [20] recently reported the CrIs of native SB, microwave-alkali pretreated SB, and enzyme hydrolysed microwave-alkali pretreated bagasse as 53.44%, 65.29% and 58.58% respectively.

### Solid-state ^13^C NMR spectroscopy

**Figure 4:**
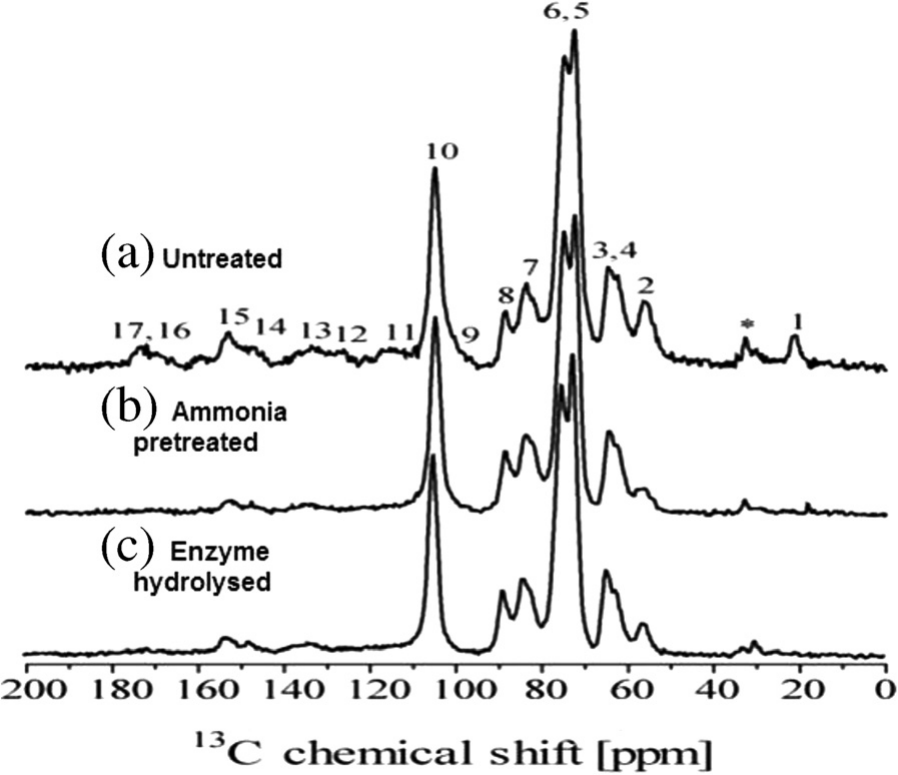
**CPMAS-TOSS NMR spectra of sugarcane bagasse. (a)** Untreated; **(b)** bagasse treated with ammonium hydroxide (20% v/v NH_4_OH, 70°C, 24 h) and **(c)** delignified bagasse after enzymatic hydrolysis. The spectra were normalized by the intensity of line 10 (C1 carbon of cellulose). CPMAS-TOSS: cross polarization under magic angle spinning with total suppression of spinning sidebands; NMR: nuclear magnetic resonance. * represents the peak which arises due to aliphatic lignin carbons not bound to oxygen.

Figure 4 shows CPMAS-TOSS spectra of the solid fractions of untreated, SCAA-SB and enzymatically hydrolysed samples. The carbon chemical shift assignments were obtained from comparison with the ^13^C NMR spectra from wood samples [25,26] and acid-alkaline pretreated sugarcane bagasse [17] .

For untreated SB sample, the lines in the 50 to 120 ppm region are primarily due to cellulose carbons with contributions from lignin and hemicellulose signals. C6 and C4 carbon from amorphous cellulose [27] are lines 3 at 63 ppm and 7 at 84 ppm, respectively, while C6 and C4 carbon in crystalline cellulose are lines 4 at 65 ppm and 8 at 88 ppm [25-27]. Lignin signals are scattered throughout all spectral regions, lines labeled as 2, 11, 12, 13, 14 and 15 in Figure 4a. For lignin signals [25,27], the line marked by * is due to aliphatic lignin carbons not bound to oxygen [27]. Hemicelluloses carbons contribute to lines, 1, 3, 6, 7, 9 and 17 [25]. Figure 4b shows the spectrum of ammonia-treated bagasse which reveals the intensity decrease of lines 1 and 17, show the removal of hemicellulose. Since all spectra were normalized with respect to line 10 (C1 carbon of cellulose), the intensity decrease of lines *, 2, 11,12,13,14 and 15 indicates a reduction of the lignin to cellulose fraction in the bagasse sample after the ammonia treatment. Figure 4c shows the spectrum of the solid fraction obtained after enzyme hydrolysis of the ammonia treated bagasse sample. Assuming that the relative amount of lignin is constant during the enzyme hydrolysis, the increase of lignin signals, lines *,2,11,12,13,14 and 15, relative to cellulose ones, lines 3,4,5,6,7,8 and 10, is associated to the removal of cellulose [17,25].

### Ethanol fermentation (SHF)

**Figure 5:**
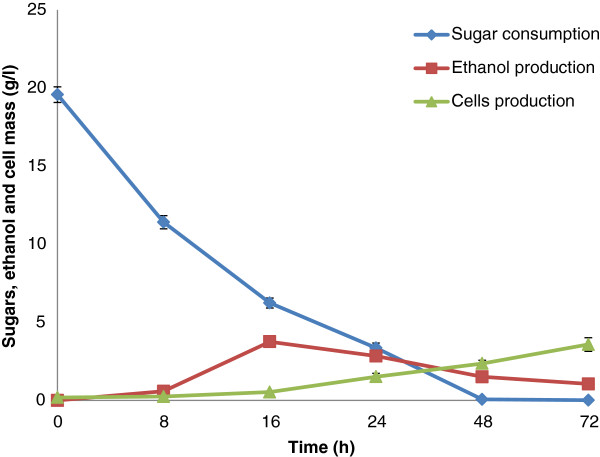
**Fermentation of enzymatic hydrolysates via separate hydrolysis and fermentation (SHF) using *S*. *stipitis *NRRL Y-7124 at 30°C and pH 5.0.**

For fermentation of enzymatic hydrolysates, a native pentose and hexose sugars fermenting yeast, *S. stipitis* NRRL Y-7124 was employed under batch cultivation conditions. *S. stipitis* has the ability to ferment pentose and hexose sugars in addition to cellobiose [28,29]. It is prudent to use a microorganism for ethanol production from lignocellulose hydrolysate that can equally convert pentoses and hexoses into ethanol with satisfactory yields and productivities [8,12]. This is because ammonia pretreated sugarcane bagasse liberates significant quantities of pentose sugars in addition to glucose and oligomers. Figure 5 shows the profiles of ethanol, cell growth and sugar consumption during the bioconversion of enzymatic hydrolysates by *S. stipitis*. It is evident, that maximum fermentation occurred within the first 24 h. Maximum ethanol production (3.83 g/l) and sugar consumption was recorded at 16 h of incubation. However, cell growth continued until the completion of fermentation at 72 h. Table 4 shows the kinetic parameters for ethanol production by *S. stipitis* NRRL Y-7124. The initial concentration of sugar present in hydrolysate before microbial fermentation was 19.52 g/l. A maximum ethanol production of 3.83 g/l was observed with ethanol yield of 0.289 g/g, productivity of 0.239 g/l/h and ethanol fermentation efficiency of 56.80%. Cells production increased continuously until the completion of the fermentation cycle. Maximum cell production of 3.57 g/l and yield of 0.18 g/g was observed. In the present study, we obtained less ethanol yield compared to previous studies. This lower ethanol yield is likely due to a lower initial concentration of sugars in the hydrolysate. Saracoglu and Arslan [29] observed 7.8 g/l ethanol production with a yield of 0.44 g/g from corn cob hemicellulosic hydrolysates by *S. stipitis* NRRL Y-7124. Nigam [30] reported ethanol production at 18.0 g/l and yield of 0.35 g/g from *Eichhornia crassipes* hemicellulosic hydrolysate when fermented with *S. stipitis* NRRL Y-7124.

**Table 4:** **Kinetic parameters for ethanol production by *S. stipitis *NRRL Y-7124 under SHF and SSF process after 72 h and 96 h respectively**

**Parameters**	**SHF**	**SSF**
Initial sugars (g/L)	19.52	27.95
Ethanol produced (g/L)	3.83	10.31
Cells produced (g/L)	3.57	n.d
Ethanol yield (g/g)	0.289	0.387
Fermentation efficiency (%)	56.80	75.88
Cells yield (g/g)	0.18 g/g	n.d
Ethanol productivity (g/L/h)	0.239	0.214
Cells productivity (g/L/h)	0.0495	n.d

n.d: not detected (Cells production was not detected in SSF). Fermentation was carried up to 72 h in SHF and 96 h in SSF. In SHF, maximum ethanol production (3.83 g/L) was observed after 16 h followed by a gradual downfall in conjunction with regular hike in cell mass production. After 16 hrs of fermentation, 13.32 g/l sugar was consumed. Ethanol yield, productivity and fermentation efficiency was calculated taking 16 hrs into consideration. Maximum cell mass (3.57 g/L) was recorded after 72 h. In SSF, yeast inoculum was added after 24 h of enzymatic saccharification (Pre-hydrolysis) and maximum ethanol production was obtained after 48 h of incubation. In SSF, after 48 hrs of fermentation, 26.6 g/l sugar was consumed by the yeast. Ethanol yield, productivity and fermentation efficiency was calculated taking 48 hrs into consideration. Fermentation efficiency (%) was calculated as the percentage of the maximum theoretical ethanol yield (0.51 g ethanol/g sugar used). The values are mean of three replicates. Standard deviation was within 10%.

### Simultaneous saccharification and fermentation (SSF)

**Figure 6:**
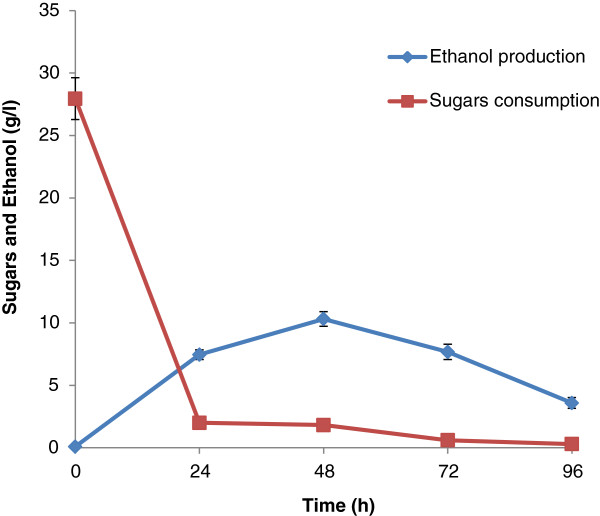
**Simultaneous saccharification and fermentation (SSF) of ammonia pretreated sugarcane bagasse using *S. stipitis *NRRL Y-7124 at 30°C and pH 5.0.** Pre-hydrolysis of ammonia pretreated SB was carried out for 24 h to initiate the enzymatic saccharification. After, 24 h of pre-hydrolysis, fermentation medium ingredients and inoculum was added to start SSF for ethanol production.

Apart from lignin removal during SCAA pretreatment of SB, part of the lignin relocates on the surface of SB increasing enzyme assisted digestibility, nutrient value and fermentability of the substrate [31,32]. These characteristics are very important for the implication of delignified substrates into SSF. Figure 5 shows the SSF of ammonia pretreated sugarcane bagasse using *S. stipitis* NRRL Y-7124. As can be seen in Figure 6, ethanol production in SSF was greater compared to SHF. Lee et al. [33] also found higher ethanol production during SSF with *S. stipitis* CBS 6054 than SHF of oxalic acid pretreated corn cob.

In the present study, after 24 h of pre-hydrolysis with cellulases, sugar release was 27.95 g/l. The yeast was added after the 24 h pre-hydrolysis step to initiate ethanol fermentation under SSF. Pre-hydrolysis has been shown to increase sugar recovery prior to ethanol production. Jin et al. [31] reported improved ethanol metabolic yield (90.3%) after 24-h of pre-hydrolysis compared to 6-h of pre-hydrolysis (88% ethanol metabolic yield) from ammonium hydroxide pretreated corn stover. In the present study, maximum ethanol production (10.31 g/l, yield 0.387 g/g and 75.88% ethanol fermentation efficiency) was observed at 48 h of incubation followed by a gradual decrease in sugar level. Maximum ethanol productivity (0.214 g/l/h) was noticed during SSF. Previously, we reported ethanol production (15.73 ± 0.44 g/l) with the yield (0.42 ± 0.02 g/g) and productivity (0.218 ± 0.04 g/l/h) after the SSF of ammonia pretreated *Saccharum spontaneum* with *S. stipitis* NCIM 3498 [8]. Cell growth during SSF was not detected due to the presence of solid substrates with the cells. Table 4 shows the kinetic parameters for ethanol production by *S. stipitis* NRRL Y-7124 under SSF process after 96 h of incubation. It is difficult to compare the results from previous studies due to difference in pretreatment conditions, enzymes used for hydrolysis, variation in fermentation conditions and microorganisms used for ethanol production.

## Conclusions

Among the conditions studied for delignification of SB, we found pretreatment at 20% (v/v) NH_4_OH at 70°C for 24 h showed maximum lignin removal (41.51%) in addition to hemicellulose loss (68.75%) which increased the cellulose amount (62.2%) in the pretreated material. SCAA-SB showed maximum reducing sugars (28.43 g/l, 0.57 g/g bagasse) after 72 h of enzymatic hydrolysis. Temperature and pretreatment time contributed the maximum influence on enzymatic hydrolysis of ammonia hydroxide pretreated SB. SEM analysis indicated the cell wall disruption and heterogeneous nature of SB after delignification and enzymatic hydrolysis. XRD analysis also showed the change in crystallinity of SCAA-bagasse and enzymatically digested SB. Solid-state ^13^C NMR spectroscopy revealed changes in carbon chemical shift assignments in lignin, hemicellulose and cellulose fractions of bagasse. When SHF and SSF of pretreated bagasse was conducted using *S. stipitis* NRRL Y-7124, 3.83 g/l yield 0.289 g/g and 56.80% ethanol fermentation efficiency) and (10.31 g/l, yield 0.387 g/g and 75.88% ethanol fermentation efficiency) was obtained respectively.

## Material and Methods

### Preparation of raw substrate

Sugarcane bagasse was acquired from Usina Vale do Rosário (Morro Agudo, S.P), air-dried, and knife milled (Marconi Equipamentos, Model No. MA 680, Piracicaba-S.P, Brazil) and passed through a 20-mesh sieve. It was washed with running water to remove dust and dried at 45°C for further experiments.

### Ammonia pretreatment optimization

For the pretreatment, sugarcane bagasse was treated with various concentration of aqueous ammonia in screw-capped laboratory bottles (Pyrex bottles) with no agitation. Solid-to-liquid ratio of 1:10 was applied. Taguchi experimental design (Taguchi method, L_8_ orthogonal array) was used to optimize the maximum delignification considering three independent variables (ammonia concentration, temperature and time) at two levels with center point (Table 1). A total number of 9 experiments were carried out in duplicate with center point (Table 2). The delignified SB obtained in each experiment was subjected to enzymatic hydrolysis and the sugars released after enzyme hydrolysis was the responsive variable (Table 2).

The experimental results were analyzed to extract independently the main effects of the factors; the analysis of variance (ANOVA) was then applied to determine which factors were statistically significant. The controlling factors were identified with the magnitude of effects qualified and the statistically significant effects determined. Accordingly, the optimal conditions were determined by combining the levels of factors that had the highest main effect value. All calculations were performed using software (Statistica 10, USA).

### Enzymatic hydrolysis

SCAA-bagasse samples were enzymatically hydrolysed to deploymerise the available carbohydrate into simpler sugars. Enzymatic hydrolysis of pretreated bagasse was performed in 150 mL Erlenmeyer flask containing (2 g d.wt. of ammonia pretreated bagasse) and 40 mL of citrate buffer (50 mM, pH: 4.8). Substrates with buffer were pre-incubated at room temperature for 1 h. Sodium azide was also added at a concentration of 0.005% to restrict any microbial growth during the course of enzymatic hydrolysis. The substrate soaked in citrate buffer was supplemented with cellulase loadings (15 FPU (Filter Paper Activity)/g of the dry biomass from Celluclast 1.5 L and 17.5 IU/g of the dry biomass of β-glucosidase from Novozym 188). Enzymatic hydrolysis was performed at 50°C at 150 rpm in incubator shaker (Innova 4000 Incubator Shaker, New Brunswick Scientific, Enfield, CT, USA). These enzymes were procured from Sigma Aldrich, USA. The enzymatic hydrolysis was performed up to 96 h. Samples were collected after every 24 h, centrifuged and analyzed to determine the total reducing sugars released.

### Separate hydrolysis and fermentation (SHF)

#### *Microorganism and inoculum preparation*

*Scheffersomyces stipitis* NRRL Y-7124, native xylose-fermenting yeast, was used for the fermentation of enzymatic hydrolysates. Strains were maintained on YPD plates and stored at 4°C. Cells were grown in 150 mL Erlenmeyer flasks containing 50 mL of YPD (10 g/l yeast extract, 10 g/l peptone, 20 g/l glucose) in an orbital shaker incubator at 30°C, shaken at 200 rpm. Following 24 h growth, broth was centrifuged and inoculum was prepared corresponding to 1.0 g/l cells (d. wt). Inoculum was aseptically transferred into enzymatic hydrolysate (50 mL) supplemented with medium ingredients defined by Canilha et al [34].

#### *Fermentation*

Fermentation of enzymatic hydrolysates (50 mL) obtained from SCAA-SB (20% v/v NH_4_OH, 24 h, 70°C) was carried out in 150 mL Erlenmeyer flasks. The flasks were maintained in a rotator shaker (Innova 4000 Incubator Shaker, New Brunswick Scientific, Enfield, CT, USA) at 30°C and 200 rpm for 72 h. Four samples were collected in first 24 h (0, 8, 16 and 24 h) followed by every 24 h until 72 h of incubation.

#### *Simultaneous saccharification and fermentation (SSF)*

SSF was performed in 150 mL Erlenmeyer flasks containing 50 mL of fermentation medium prepared as above. For saccharification of SCAA-SB (20% v/v NH_4_OH, 24 h, 70°C), an appropriate amount of FPase and β-glucosidase was added as mentioned above. Sodium azide was not added in SSF. During first 24 h, enzymatic hydrolysis was carried out at 50°C and 150 rpm in a rotator shaker (Innova 4000 Incubator Shaker, New Brunswick Scientific, Enfield, CT, USA). After 24 h of enzymatic hydrolysis, *S. stipitis* was inoculated at a cell concentration of 1.0 g/l (d.wt). This reaction was carried out at 30°C, 200 rpm for 96 h.

### Characterization of native, SCAA-SB and EH-SB

#### *Cell wall compositional characterization*

Native and SCAA-SB (20% v/v NH_4_OH, 24 h, 70°C) was characterized for moisture, lignin (Klason and acid soluble), hemicellulose and cellulose content following the method of Gouveia et al [35].

#### *Scanning electron microscopy (SEM)*

The SEM analysis of native, SCAA (20% v/v NH_4_OH, 24 h, 70°C) and enzymatically hydrolysed SB was performed as described earlier [19]. Native, SCAA and enzymatically hydrolysed SB distributed on a 12 mm glass cover slip coated with poly-L-lysine (Sigma Diagnostics, S.P. Brazil). The dried section was mounted on aluminum stubs, sputter-coated (JEOL JFC-1600) with a gold layer, and used for scanning. The prepared samples were scanned and imaged with a Hitachi S520 scanning electron microscope (Hitachi, Tokyo, Japan).

#### *X-ray diffraction* (*XRD*)

The crystalline nature of native, SCAA-treated (20% v/v NH_4_OH, 24 h, 70°C) and enzyme digested SB was analyzed by using a in a Rigaku Rotaflex diffractometer model RU200B (Tokyo, Japan) using monochromatic CuKa radiation (1.54 Å) set at 40 KV, 30 mA. The goniometer scanned a 2θ range between 5° and 65° at a 2°/min scanning rate. Samples were scanned over the range of 100 <2θ <500 with a step size of 0.05° and the crystallinity index (CrI) were determined using the empirical method proposed by Segal et al. [21] and Park et al. [22]. Samples were measured in duplicates and the average values of CrI was obtained from the relationship between the intensity of the 002 peak for cellulose I (I*002*) and the minimum dip (I*am*) between the 002 and the 101 peaks following the formula:

$$\mathrm{CrI}=\frac{I\;002-I\;\mathrm{amorphous}\;}{I\;002}\times 100\%.$$

### Solid-state ^13^C nuclear magnetic resonance (NMR) spectroscopy

Solid-state ^13^C NMR analyses of native, SCAA-SB (20% v/v NH_4_OH, 24 h, 70°C) and enzyme digested SB were performed using a Varian Inova spectrometer operating at ^13^C and ^1^H frequencies of 88.02 and 350.50 MHz, respectively [17,36]. A Varian 7-mm magic-angle spinning MAS double-resonance probe head was used. Spinning frequencies of 4.5 kHz were controlled by a Varian pneumatic system that ensures a rotation stability of about 2 Hz. Ramped cross-polarizations under magic angle spinning (CPMAS) combined with total suppression of spinning sidebands (TOSS) and heteronuclear ^1^H decoupling (CPMAS-TOSS) was used to acquire the ^13^C spectra. Typical π/2 pulse lengths of 4.0 μs (^13^C) and 4.5 μs (^1^H), cross-polarization time of 1 ms, acquisition time of 30 ms, and recycle delays of 2 s were used in all NMR analysis.

### Analyses

Total reducing sugars (TRS) were estimated by spectrophotometer (Beckman Du 640 B, USA) following the DNS method of Miller [37]. Ethanol production was analyzed by HPLC (Waters) using a refraction index detector and a Biorad Aminex HPX-87H column at 45°C. Sulfuric acid (0.01 N) at a flow rate of 0.6 mL/min was used as an eluent and the injection volume was of 20 μL. Cell concentrations were determined in SHF by turbidimetry using the spectrophotometer (Beckman DU640B, USA). However, cell concentrations were not measured in SSF due to the presence of bagasse particles and enzymes in fermentation broth. The absorbance measurements at 600 nm were correlated with the cell concentrations (g/l) following the calibration equation:

y=1.0804x+0.006.

## Abbreviations

SCAA: Soaking in concentrated aqueous ammonia; SAA: Soaking in aqueous ammonia; SB: Sugarcane bagasse; AFEX: Ammonia fiber expansion; SCAA-SB: Soaking in concentrated aqueous ammonia- sugarcane bagasse; SEM: Scanning electron microscopy; XRD: X-ray diffraction; NMR: Nuclear magnetic resonance; CPMAS-TOSS: Cross-polarization under magic angle spinning and total suppression of spinning sidebands; FPU: Filter paper units; CBU: Cellobiase units; SHF: Separate hydrolysis and fermentation; CrI: Crystallinity index; SSF: Simultaneous saccharification and fermentation; YPD: Yeast peptone dextrose; HPLC: High performance liquid chromatography.

## Competing interests

The authors declare that they have no competing interests.

## Authors’ contributions

AKC planned and performed the biomass pretreatment, enzymatic hydrolysis, ethanol fermentation, as well as the analysis of the results and manuscript writing. FAFA assisted in biomass characterization and fermentation experiments. MBS analyzed all the Taguchi optimization results and reviewed the manuscript draft. SSS coordinated the overall study, analysis of results and finalizing the manuscript. All authors suggested modifications to the draft and approved the final manuscript.
